# Transcription factor motif enrichment in whole transcriptome analysis identifies STAT4 and BCL6 as the most prominent binding motif in systemic juvenile idiopathic arthritis

**DOI:** 10.1186/s13075-018-1603-2

**Published:** 2018-05-30

**Authors:** Boris Hügle, Anastasia Schippers, Nadine Fischer, Kim Ohl, Bernd Denecke, Fabio Ticconi, Bas Vastert, Ivan G. Costa, Johannes-Peter Haas, Klaus Tenbrock

**Affiliations:** 1 German Center for Pediatric and Adolescent Rheumatology, Gehfeldstrasse 24, 82467 Garmisch-Partenkirchen, Germany; 2 Department of Pediatrics, Universitätsklinikum Aachen, Aachen, Germany; 3 IZKF Research Group Bioinformatics, RWTH Aachen Medical Faculty, Aachen, Germany; 4 University Medical Center Utrecht, Utrecht, Netherlands

**Keywords:** Juvenile systemic arthritis, Juvenile idiopathic arthritis, RNA expression, HLA-DRB1, CD74, CD177

## Abstract

**Background:**

The term systemic juvenile idiopathic arthritis (sJIA) describes an autoinflammatory condition characterized by arthritis and severe systemic inflammation, which in later stages can transform into interleukin (IL)-17-driven autoimmune arthritis. IL-1 antagonists have been used with good efficacy in the early stages of sJIA.

**Methods:**

A whole transcriptome analysis of peripheral blood RNA samples was performed in six patients with sJIA and active systemic disease, before initiating treatment with the IL-1β receptor antagonist anakinra, and after induction of inactive disease, compared with a single-sample control cohort of 21 patients in several clinical stages of sJIA activity. Whole transcriptomes were compared longitudinally and interindividually including gene ontology and motif enrichment analysis of differentially expressed genes.

**Results:**

There were 741 transcripts were identified using a threshold with a *p* value <0.01 and a fold change > 2. HLADRB1 and CD74 were identified as the most strongly upregulated genes in inactive compared to active disease; CD177 expression was significantly enhanced in active disease compared to inactive disease. Motif enrichment analysis revealed STAT4, BCL6, and STAT3 as the most prominent transcription factors that were present during active disease. In addition, strong upregulation of the major histocompatability complex II (MHCII) ligand CD74 was found in both active and inactive sJIA compared to healthy controls.

**Conclusion:**

Using transcription factor motif enrichment, this study identifies novel putative pathways in sJIA (STAT4, BCL6) implicating B cell activation at an earlier stage than predicted in refractory disease. The implication of BCL-6 dependent pathways argues for occurrence of autoimmunity early within the process of sJIA chronification. Transcriptional regulation of HLA-DRB1, a recently described independent genetic risk factor, in combination with its cooperating partner CD74 in patients where sJIA is confirmed, supports pathogenic involvement in alterations in antigen presentation during sJIA.

**Electronic supplementary material:**

The online version of this article (10.1186/s13075-018-1603-2) contains supplementary material, which is available to authorized users.

## Background

Systemic juvenile idiopathic arthritis (sJIA) is an inflammatory disease with severe systemic inflammation leading to marked morbidity and mortality. Children with sJIA usually present with fever, arthritis, and a typical rash and can have a highly variable outcome [1]. Despite sJIA being listed as a subtype of juvenile idiopathic arthritis, it is currently considered to be of autoinflammatory rather than autoimmune origin [2, 3]. At least two phenotypes of sJIA can be identified by their clinical course: patients with monocyclic sJIA with only a single phase of severe systemic inflammation and concomitant arthritis, and patients developing chronic disease with recurrent flares of autoinflammation and severe arthritis [1].

Peripheral blood mononuclear cells (PBMC) and monocytic cells from patients with active sJIA have been analyzed in gene expression studies and have been shown to have a variable RNA expression pattern in patients with sJIA compared to healthy controls, patients with other autoimmune diseases, and patients with other subtypes of JIA, with uneven results [4, 5]. With the exception of a recent study looking at RNA expression in patient samples from two clinical trials of canakinumab, these studies have so far only used a cross-sectional approach, and patients with sJIA were not stratified according to their disease activity [6].

The objective of this study was to perform longitudinal whole transcriptome analysis of children with sJIA refractory to conventional, non-biological therapy before treatment with IL-1 antagonists and after achieving inactive disease, to identify transcriptional patterns and possible novel markers involved in the pathogenesis of the disease.

## Methods

### Patients

Clinical data and patient samples were acquired from the AID-Net database, a German registry and biobank that prospectively collects information and biomaterials from patients with autoinflammatory syndromes including periodic fever syndromes and sJIA. Written informed consent was obtained from the patients prior to inclusion. Healthy aged-matched controls undergoing elective surgery were recruited from the pediatric department of the university hospital RWTH Aachen. Further information on data safety and pseudonymization has been published previously [7]. A single-center sample of all patients with sJIA at the German Center for Pediatric and Adolescent Rheumatology was obtained between January 2010 and March 2015. All patients who met the following criteria were included in cohort I, which was set up for longitudinal analysis: (1) confirmed diagnosis of sJIA according to International League of Associations for Rheumatology (ILAR) criteria [3], (2) initial treatment with IL-1 antagonists after persistence of symptoms while taking non-steroidal antirheumatic drugs and/or corticosteroids, (3) achievement of subsequent inactive disease, and (4) available samples from at least two time points, i.e. at initial manifestation prior to treatment with IL-1 antagonists, and at a point of inactive disease on treatment with IL-1 antagonists. Additional samples from recurring disease flares and subsequent periods of inactivity were also acquired, where available.

A second cohort, cohort II, was set up for verification purposes. These were patients with only a single sample available, who had active systemic disease, active polyarticular disease without systemic activity or inactive disease.

Inactive disease was defined according to the American College of Rheumatology provisional criteria [8]. Active systemic disease was defined as presence of fever, rash, serositis, splenomegaly, or generalized lymphadenopathy attributable to JIA, with or without concurrent arthritis at that time. Active polyarticular disease was defined as presence of arthritis without fever, rash, serositis, splenomegaly, or generalized lymphadenopathy.

### Data collection

A retrospective chart survey was used to extract demographic data, including date of first manifestation, date of diagnosis, total joint count at diagnosis, laboratory parameters at diagnosis including C-reactive protein, ferritin and thrombocyte count and the initial dose of prednisolone and anakinra.

### Sample preparation

Whole blood was drawn during active disease before start of treatment with anakinra, after achievement of inactive disease and on subsequent visits. Peripheral blood was collected using PAXgene Blood RNA tubes (Qiagen, Valencia, CA, USA). RNA was extracted at the collection site using RNeasy columns (Qiagen, Valencia, CA, USA), then stored at − 20 °C. RNA quantity and quality was assessed using a Pico100 Picodrop ul Spectrophotometer (Picodrop, Saffron Walden, UK).

### Expression analysis

Genome-wide transcriptome analyses were performed in cohort I using Gene Chip® Human HTA 2.0 arrays (Affymetrix, Santa Clara, CA, USA). Prior to analysis, RNA quality was assessed using the RNA 6000 Nano Assay with the 2100 Bioanalyzer (Agilent, Santa Clara, CA, USA). Samples for the HTA 2.0 arrays were prepared and hybridized to the arrays according to the Affymetrix WT Plus Kit manual. Briefly, for each sample, 100 ng of total RNA was reverse transcribed into complementary DNA (cDNA) using a random hexamer oligonucleotide tagged with a T7 promoter sequence. After second-strand synthesis, double-strand cDNA was used as a template for amplification with T7 RNA polymerase to obtain antisense cRNA. Random hexamers and deoxyribose adenine triphosphates (dNTPs) spiked out with deoxyuridine triphosphate (dUTP) were then used to reverse transcribe the cRNA into single-stranded sense strand cDNA. The cDNA was then fragmented with uracil DNA glycosylase and apurinic/apyrimidic endonuclease 1. Fragment size was checked using the 2100 Bioanalyzer and ranged from 50 to 200 bp. Fragmented sense cDNA was biotin-end-labeled with terminal deoxynucleotidyl transferase (TdT) and probes were hybridized to the Gene 2.0 arrays at 45 °C for 16 h with 60 rpms. Hybridized arrays were washed and stained on a Fluidics Station 450 (program FS450 0002) and scanned on a GeneChip® Scanner 3000 7G (both Affymetrix). Raw image data were analyzed with Affymetrix® Expression ConsoleTM Software (Affymetrix, USA), and gene expression intensities were normalized and summarized with a robust multiarray average algorithm [9]. Transcripts that were expressed differently more than twofold with a raw *p* value <0.01 between the sample groups were categorized as regulated. Enrichment analysis for Wiki pathways was performed using WebGestalt [10]. For the enrichment analysis only genes that changed at least twofold with a *p* value <0.01 between patients with active disease and those with inactive disease were taken into consideration.

### Reverse transcription-polymerase chain reaction (RT-PCR)

For verification purposes, RT-PCR for several genes was performed in cohort I and II. The genes selected were chosen both due to the results of the expression analysis and previous descriptions in the literature [6, 11]. cDNA was generated from RNA using RevertAid H Minus First Strand cDNA Synthesis Kit (Thermo Fisher Scientific, USA) according to the manufacturer’s instructions. Standard real-time PCR was carried out on TaqMan with the ABI prism 7300 real-time PCR systems (Applied Biosystems by Life Technologies, Germany) using the DNA intercalating dye SYBR Green Kit (Eurogentec, Germany). The housekeeping gene used was ribosomal protein L (RPL). The following primer sequences were used: for HLA-DRB1, TTC TTC AAT GGG ACG GAG CG (forward) and TTC CAG TAC TCA GCG TCA GG (reverse); for CD74, TTA TCT CCA ACA ATG AGC AAC T (forward) and ACA GGA AGT AGG CGG TGG T (reverse); for CD177, CAT GTG TGG AAG GTG TCC GA (forward) and CTT GGG GTC CGC TCT CAA TG (reverse); and for RPL, AGGTATGCTGCCCCACAAAAC (forward), TGTAGGCTTCAGACGCACGAC (reverse).

The relative quantification method was applied and delta cycle threshold (ΔCt) values were determined by subtracting the Ct of the housekeeping gene (RPL) from the Ct of the target gene for each sample, respectively. Fold change was compared in active disease and inactive disease in the same individual using the ∆Ct method.

### Statistical analysis

Clinical data were analyzed using descriptive statistics. Statistical analysis was performed using SPSS version 21.0 (SPSS Inc., Chicago, USA). Microarray data were imported into GeneSpring GX 7.3.1 software (Agilent Technologies, Santa Clara, USA) and preprocessed using robust multichip analysis (RMA), followed by normalization of each probe to the median of all samples. Distance-weighted discrimination was used to align the centroids of predefined groups (12–16) to control for batch-to-batch variation.

Gene Ontology (GO)-based analysis of biological process was performed using AltAnalyze 2.1.0 software (altanalyze.org); significance values were between an adjusted *p*-value of 3.17e-08 and 0.0005.

#### Motif enrichment analysis

Transcription factor motif enrichment on upregulated/downregulated genes was performed using MotifMatch (www.regulatory-genomics.org). In short, this software searches for binding sites on the promoter region of all candidate genes (1 kbp upstream). Motifs were obtained from the Jaspar database. It then performs a Fisher exact test to evaluate if the proportion of binding sites in the gene sets is higher than expected by chance. The *p*-values were adjusted for multiple testing using the Benjamini-Hochberg method.

## Results

### Study population

Cohort I included longitudinal samples of six children with sJIA, with all patients having at least a sample pair prior to treatment with anakinra and with inactive disease on anakinra; samples were also available from two patients during a flare after withdrawal of anakinra. Cohort II consisted of single samples from eight patients with systemically active sJIA, five patients with sJIA with a polyarticular flare but no clinical signs of systemic activity, and eight patients with inactive sJIA. The clinical and demographic data at time of diagnosis in both cohorts are given in Table 1. All patients were of Caucasian origin and initially showed a typical clinical picture of sJIA with rash, fever, and arthritis, and typical bloodwork with elevated inflammatory markers. All patients in cohort I reacted rapidly to treatment with IL-1 antagonists, achieving an inactive disease state within days to weeks.

**Table 1:** Demographic, clinical and laboratory characteristics of the study cohorts

Patient	Cohort I (*n* = 6)	Cohort II (*n* = 8) Active disease, systemic	Cohort II (*n* = 5) Active disease, polyarticular	Cohort II (n = 8) Inactive disease
Gender	5 male, 1 female	5 male, 3 female	2 male, 3 female	5 male, 3 female
Age at diagnosis, years (median, range)	5.3 (1.8–12.9)	6.8 (0.4–17.4)	6.0 (0.5–15.3)	3.5 (0.4–9.7)
Time since first symptoms (median, range)	71 days (47–107 days)	18.6 months (1.6–109.7 months)	86.9 months (7.4–198.3 months)	105.1 months (52.6–195.8 months)
Number of active joints (median, range)	2 (1–18)	3.5 (0–10)	3 (2–4)	0 (0–0)
Platelet count (median, range)	520,500/mm^3^ (474,000–557,000/mm^3^)	352,000/mm^3^ (151,000–649,000/mm^3^)	276,000/mm^3^ (195,000–395,000/mm^3^)	306,500/mm^3^ (208,000–394,000/mm^3^)
Rheumatoid factor, negative	6/6 (100%)	8/8 (100%)	5/5 (100%)	8/8 (100%)
Ferritin, μg/l (median, range)	414 (32–1785 )	754 (234–9980 μg/l)	n.d.	n.d.
C-reactive protein, mg/dl (median, range)	11.84 (4.87–25.6)	3.65 (1.10–26.4)	0.07 (0.03–6.57)	0.11 (0.03–0.63)
Initial prednisolone dose, mg/kg (median, range)	1.6 (0–2.08)	n.a.	n.a.	n.a.
Initial anakinra dose, mg/kg (median, range)	1.63 (1.02–2.5)	n.a.	n.a.	n.a.

*n.a.* not applicable, *n.d.* not determined

### Patients with sJIA and inactive disease have differences in RNA expression profiles compared to patients with active disease and disease flares

**Fig. 1:**
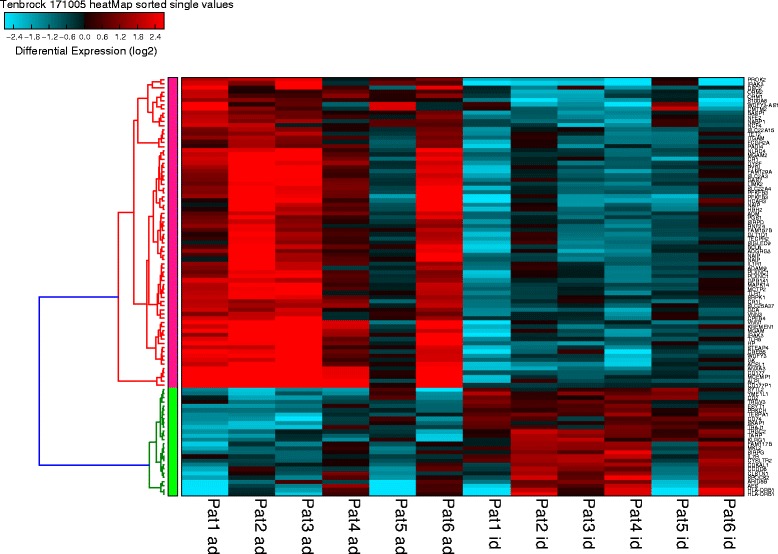
Heatmap with hierarchical clustering of patients (Pat) with active disease (ad) and inactive disease (id). Shown are all genes with fold change > 3

**Fig. 2:**
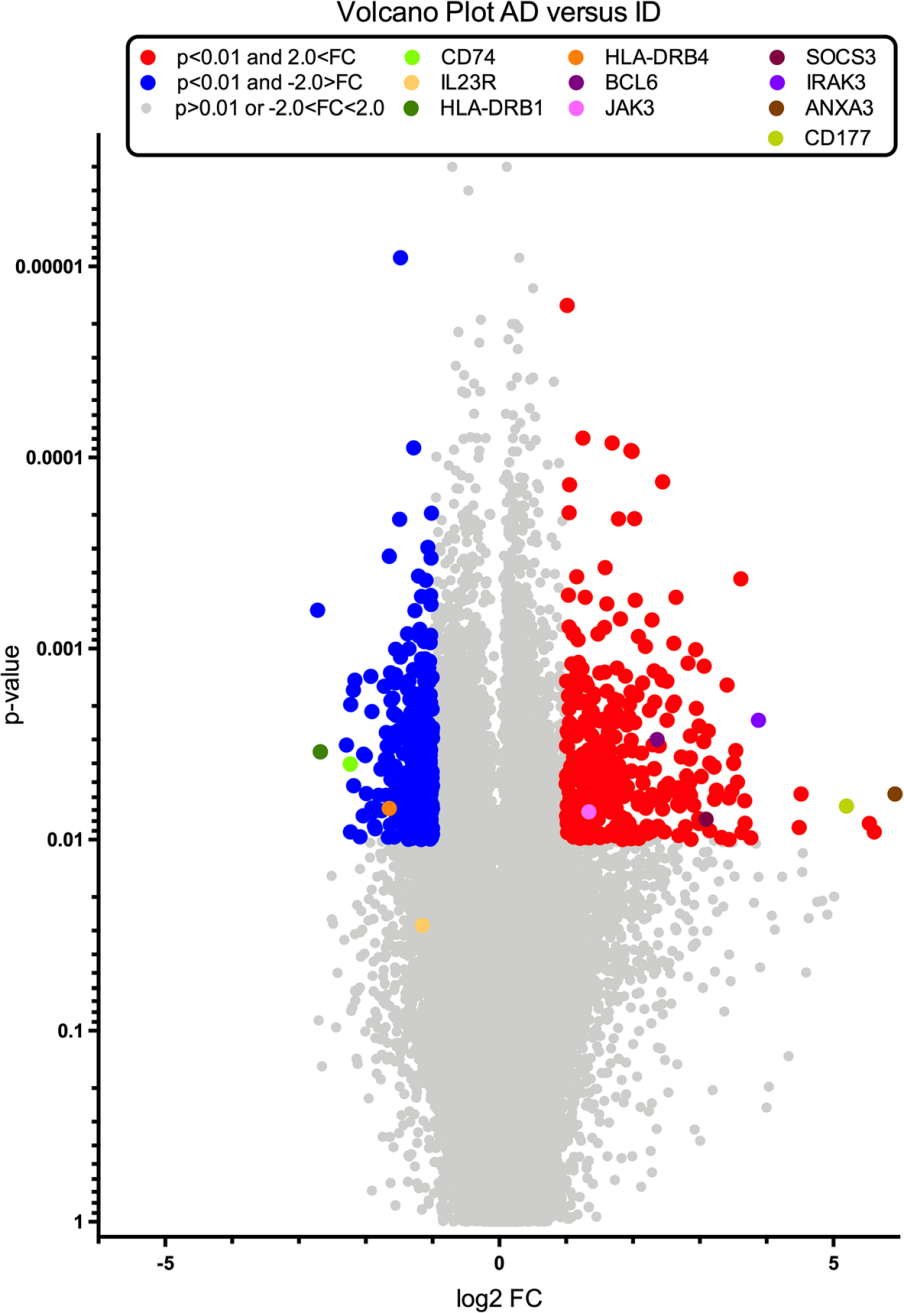
Regulated genes in patients with active disease (ad) versus inactive disease (id) using fold change (FC) > 2 and a *p* value <0.01. Red dots include genes with fold change > 2 in active disease, blue dots with a fold change <− 2. Names of highly regulated and significant genes are depicted in the graph using different colors

Using a *p* value <0.01 and fold change > 2, 741 transcripts encoding for 481 known genes were identified (Additional file 1: Table S1) that were significantly differently expressed in inactive disease compared to active disease (both on initial presentation and during disease flare), of which most were associated with immune- mediated processes (Table 2, Figs. 1 and 2). Of these, genes, 239 were downregulated while 242 were upregulated in active disease. Using fold change > 3 as a more stringent criterion, more than 100 genes still remained. Gene Ontology (GO)-based analysis favored pathways of the innate immune response as the most significantly represented pathways in active disease (Table 2). Some of the highly regulated genes (HLA-DRB1, CD74, CD177) were confirmed using RT-PCR, as described below. Additional data on ANXA3/annexin A 3, a gene locus where a SNP within the gene has been identified as a risk factor in rheumatoid arthritis, and IL-1 receptor associated kinase 3 (IRAK3), are presented in Additional file 2: Figures S1 and S2 [12].

**Table 2:** Ontology-based analysis of the most significantly regulated genes

Inflammatory response (GO:0006954)	
Regulation of T cell differentiation (GO:0045580)	
Acute inflammatory response (GO:0002526)	
Positive regulation of T cell differentiation in thymus (GO:0033089)	
Response to molecule of bacterial origin (GO:0002237)	
Regulation of lymphocyte differentiation (GO:0045619)	
Regulation of T cell activation (GO:0050863)	
Regulation of syncytium formation by plasma membrane fusion (GO:0060142)	
Positive regulation of leukocyte activation (GO:0002696)	
Positive regulation of cell activation (GO:0050867)	
Activation of innate immune response (GO:0002218)	
Response to lipopolysaccharide (GO:0032496)	
Detection of external biotic stimulus (GO:0098581)	
Activation of immune response (GO:0002253)	
Negative regulation of immune response (GO:0050777)	

Shown are the 15 biological processes that are the most stringent according to the *p* value in the Gene Ontology (GO) analysis

Of note, patient 5 had a markedly different expression pattern in active disease, and patients 2 and 5 had a different expression pattern in inactive disease (Fig. 1). Patient 5 differs to the other patients in being the only female patient. The marked difference in gene expression patterns is, however, more likely due to the fact that patient 5 received three methylprednisolone pulses prior to the RNA sample being drawn. Patient 2 also received one pulse of methylprednisolone, which the others did not.

As for the changes in inactive disease, patients 1 and 5 required ongoing IL-1 blockade and reacted with systemic signs to withdrawal, while the other patients were able to taper and discontinue the IL-1 antagonists (both anakinra and canakinumab) over the course of the next year.

### Motif enrichment analysis identifies STAT4, BCL6 and STAT3 as the most prominent binding sites during active disease

**Fig. 3:**
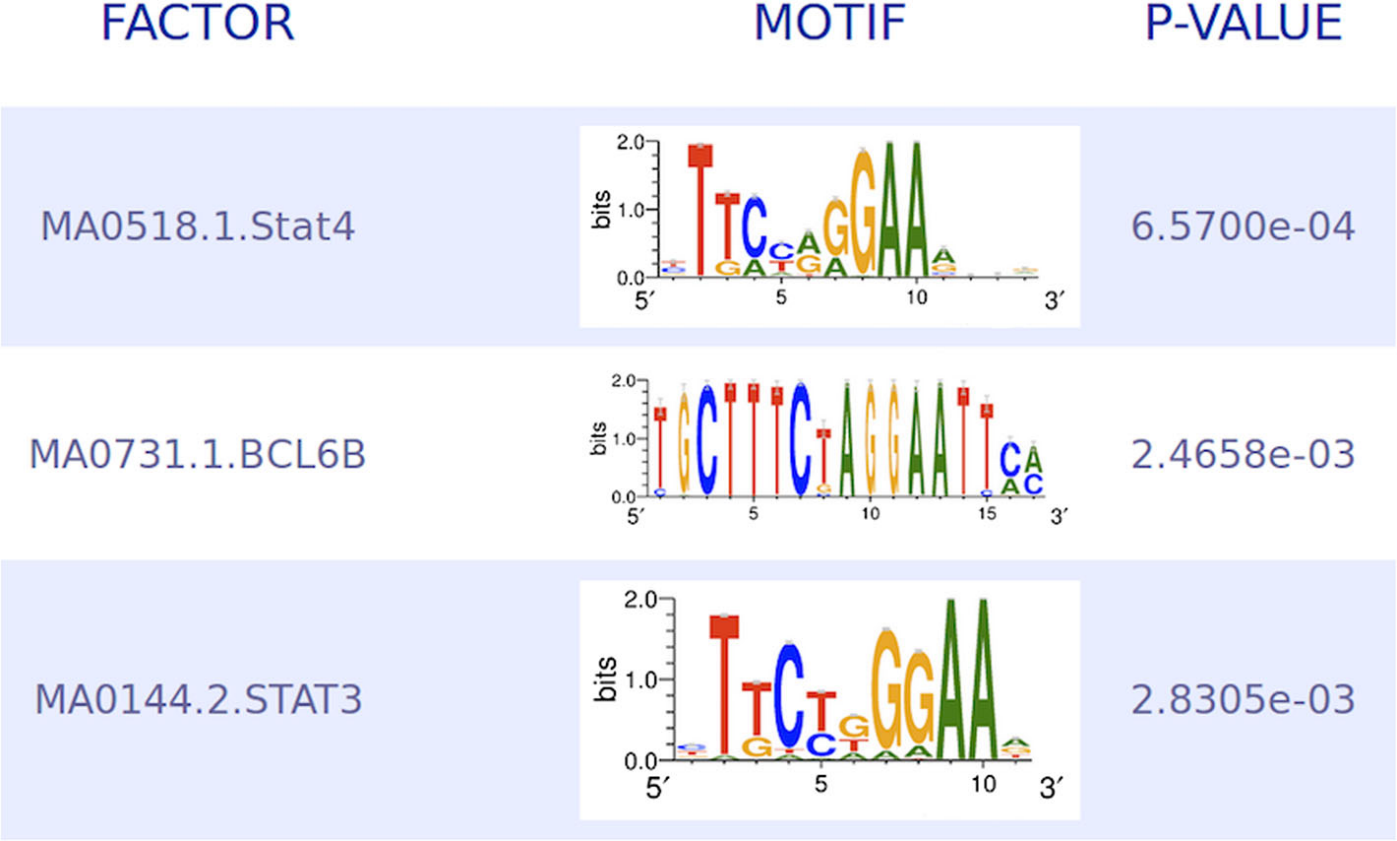
Motifs of the most regulated transcription factor binding sites in the whole transcriptome of patients with active systemic juvenile idiopathic arthritis (sJIA) compared to inactive sJIA, analyzed using MotifMatch

In order to identify pathways that might be of relevance in sJIA, we performed transcription factor (TF) motif enrichment analysis to evaluate which TFs are preferably binding in the promoter of upregulated or downregulated genes. The most prominent binding motif in upregulated genes in active sJIA was STAT4, followed by BCL6 and STAT3 (adjusted *p* value <0.005) (Fig. 3). Moreover BCL6 was also upregulated on the array in active disease (fold change 5.15, *p* = 0.002, Fig. 2), while STAT4 and STAT3 were not.

### HLA-DRB1 is upregulated in inactive compared to active disease

**Fig. 4:**
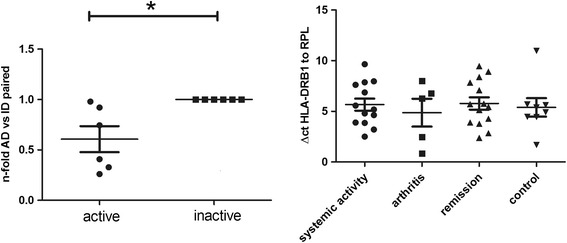
Expression of HLA-DRB1 in active (AD) versus inactive (ID) systemic juvenile idiopathic arthritis using reverse transcription PCR. Left graph, fold change (*n*-fold) of longitudinal samples before and after treatment with anakinra, with inactive disease set to level 1 (*p < 0.05). Right graph, delta cycle threshold (ΔCt) values (relative expression values related to ribosomal protein L (RPL)) in active systemic disease (cohorts I and II), active polyarticular disease, and inactive disease/remission, and controls (all cohort II)

Initially we examined transcripts shown to be regulated during sJIA, to validate that the patients under study exhibited the features described previously for this condition. For example, there was a fold change of 4.76 (*p* = 0.006) in S100A8 in our patients, as expected [13, 14]. Our analysis revealed HLA-DRB1 as the most strongly upregulated gene in inactive compared to active disease (fold change 6.8, *p* = 0.003, Fig. 2). This observation was confirmed using RT-PCR in the longitudinal per-patient sample analysis, while we did not find upregulation in cohort II compared to healthy controls due to high individual expression differences (Fig. 4). Other HLA class II genes, for example HLA-DRB4 (fold change − 2.92, *p* = 0.008, Fig. 2), HLA-DRB3 and HLA-DRB6, were found to be downregulated.

### CD74 is regulated in sJIA irrespective of disease activity

**Fig. 5:**
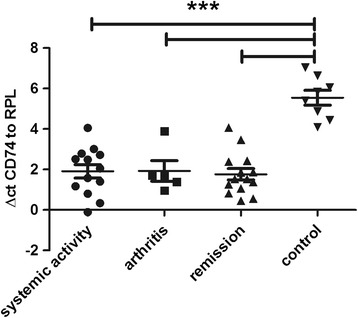
Expression of CD74 using reverse transcription PCR: delta cycle threshold (ΔCt) values (relative expression values related to ribosomal protein L) in controls, active systemic disease, active polyarticular, and inactive disease (all ***p < 0.001)

CD74 was also upregulated in inactive compared to active disease on an individual per patient base (fold change 4.73, *p* = 0.036, Fig. 2). However, and more strikingly, in cohort II CD74 was much higher expressed in patients with systemic activity as well as inactive disease compared to age-matched healthy controls using RT-PCR (Fig. 5). The protein encoded by this gene is the invariant chain of the HLA-DR complex, which is associated with class II major histocompatibility complex (MHC) including HLADRB1 and is an important chaperone that regulates antigen presentation for immune response.

### CD177 is strongly upregulated in active disease

**Fig. 6:**
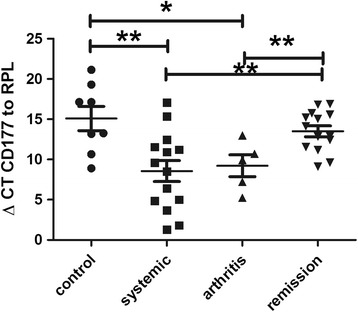
Expression of CD177 using reverse transcription PCR: delta cycle threshold (ΔCt) values (relative expression values related to ribosomal protein L) in controls, active disease, and inactive disease (***p < 0.001)

CD177 was upregulated in patients with active disease compared to those with inactive disease or healthy controls (fold change 37, *p* = 0.007, Fig. 2), which has recently been described in the study by Brachat et al. and confirms their findings using RT-PCR (Fig. 6) [6]. This gene encodes a glycosyl-phosphatidylinositol-linked cell surface glycoprotein that plays a role in neutrophil activation. The protein can bind platelet endothelial cell adhesion molecule-1 and function in neutrophil transmigration.

## Discussion

By using comparative array analysis of RNA expression in a cohort of sJIA patients in different stages of disease refractory to conventional therapy, we were able to show differential regulation of a variety of genes, comparing inactive disease on IL-1 antagonist treatment with active disease within the same patient before treatment. Regulation of specific genes has been confirmed in an independent cohort of single samples from patients with sJIA with either active or inactive disease.

Previously, a specific sJIA signature of RNA expression has been described in 44 patients with sJIA in various stages of the disease [4]. In this study, 12 patients with systemic and arthritic symptoms displayed a specific expression pattern compared to healthy controls and patients with polyarticular (non-systemic) flare. Similar results were found by Barnes et al. in a cohort of 21 patients with sJIA, which also demonstrated a specific pattern of sJIA different from other subtypes of JIA [5]. A recent study by Brachat et al. on sJIA RNA samples from two studies using canakinumab also demonstrated a large number of differentially expressed genes in patients prior to and after initiation of anti-IL-1 treatment [6]. Similar to these preceding studies, the overall specific sJIA signature consists of regulation of the inflammatory response and innate immune pathways including the IL-1 pathway, IL-6 and toll-like receptor 1, but also modulation of lymphocyte differentiation and response, with an emphasis on T cells (Table 2). A recent large genome-wide association study also demonstrated a framework of pathophysiological pathways that appears to be specific for sJIA [15].

Transcription factor motif enrichment analysis in patients with active and inactive disease identified STAT4, BCL6, and STAT3 as the most prominent motifs within the regulated genes. STAT4 is mainly expressed in myeloid cells and is the transcription factor downstream of IL-12, which has been identified as a potential biomarker in sJIA, but has not been discussed as a potential therapeutic target so far [16–18]. We have previously shown that the likelihood of the occurrence of a polymorphism enhancing IL-12 expression was somewhat higher in patients with sJIA compared to other forms of JIA, which points toward IL-12 being more prominent in sJIA than previously thought [19]. In our array analysis, IL-12 was not differentially expressed; however, the IL-23 receptor, which is involved in IL-12 downstream signaling was downregulated in active disease (fold change 2.5, *p* = 0.012, Fig. 2), which suggests a physiological reaction towards the proinflammatory state.

The identification of BCL6 as the second most prominent binding motif is more striking: BCL6 is a lineage transcription factor for follicular T helper (Tfh) cells, a cell type that is important for B cellular responses. This cell type is especially important for autoimmune arthritis mediated by gut bacteria [20]. BCL6 expression has been shown to be sustained by IL-6 signaling in patients with rheumatoid arthritis (RA), and specific targeting of IL-6 using tocilizumab in patients with RA results in a significant reduction of circulating Tfh cells; IL-21 production by Tfh cells was also correlated with reduced expression of antibody-producing plasmablasts [21]. BCL6 was also directly upregulated on the array, which supports the hypothesis that this TF is of major importance (fold change 5.15, *p* = 0.002, Fig. 2), and would also argue for even earlier involvement of autoimmunity with B cell responses in the course of the disease [22]. There is some evidence of B cell-driven autoimmunity in the later phases of sJIA, in which autoantibody production has been demonstrated [23]. Our findings here indicate that B cell activation might already be present during the first phase of disease where autoinflammation is the most prominent feature. This might potentially determine whether the patients develop a polyarticular course later. In contrast, finding STAT3 as the third most prominent transcription factor is unsurprising, since STAT3 signals downstream of IL-6, a well-known therapeutic target using tocilizumab in sJIA [24, 25]. In the study by Brachat et al., where canakinumab was used for IL-1ß blockade, IL-6 declined by day 3 and remained suppressed over time [6]. STAT3 was not differentially expressed on the array; however, STAT3 is usually activated by phosphorylation and not by transcriptional regulation. Nevertheless, IL-6 signals via JAK3, which then activates STAT3 and is downstream of the IL-6 receptor, and it was found to be enhanced (fold change 2.5, *p* = 0.006). Vice versa, the antagonist of STAT3 signaling is the suppressor of cytokine signaling 3 (SOCS3), which was highly induced in active disease (fold change 8.5, *p* = 0.012, Fig. 2), probably to counterbalance the overwhelming immune stimulation.

HLA-DRB1 and its cooperation partner CD74 were upregulated in the active disease stage, and CD74 was upregulated in the inactive disease stage in patients with sJIA in this study. Certain genotypes of HLA-DRB1, most notably HLA-DRB1*11, have been found to be strongly associated with sJIA in a large recent study of 982 patients across nine different populations, and also in a fine-mapping study of the HLA locus comparing it to other forms of JIA [11, 15, 26]. However, no expression studies of HLA-DRB1 in sJIA have been performed to date. HLA-DRB1 has also previously been demonstrated to be strongly associated with early, severe RA [27]. RA-associated HLA-DRB1 alleles have conserved amino acid sequences in position 70–74 of the molecule. This molecular structure is termed the shared epitope, with a variety of hypotheses explaining its function [28]. An association with the shared epitope has also been found in children with rheumatoid-factor positive JIA [29]. However, RNA expression patterns of the shared epitope are variable, and apparently dependent on the copy number [27].

CD74 is the invariant chain of HLA-DR and therefore a cooperation partner on the cell surface, where it critically regulates antigen presentation. It also serves as a cell surface receptor for the cytokine macrophage migration inhibitory factor (MIF) which, when bound to the encoded protein, initiates survival pathways and cell proliferation. In cooperation with IL-12, MIF is important for survival in children with malaria and trypanosoma infection [30, 31]. CD74 also interacts with amyloid precursor protein and suppresses the production of beta amyloid [32]. The high expression of CD74 even in an inactive disease state could serve as a possible candidate for disease markers for sJIA, and possibly - in the context of MIF - even for therapeutic intervention.

An additional gene that was highly upregulated in the active stages of sJIA was CD177, confirming recent results [6]. CD177 codes for human neutrophil antigen 2 (HNA-2), also called NB1, a cell surface glycoprotein [33]. HNA-2 expression is highly variable in humans and is lacking in approximately 3–5% of the North American population [34]. HNA-2 plays an important role in myeloid cell proliferation and function of neutrophils, including transendothelial migration [35]. It has also been associated with anti-neutrophil-cytoplasmic antibody (ANCA)-associated vasculitides, where CD177 has been proposed as a receptor of mPR3 on the neutrophil surface [36]. Interestingly, CD177 has also been observed as the most upregulated parameter in a microarray study of purified neutrophils from patients with septic shock. However, since we performed our analysis in whole blood, the numbers of neutrophils could also have had a considerable effect on our findings. There was, however, no difference in CD177 transcription between active systemic and active polyarticular disease, while neutrophil counts were significantly lower in polyarticular disease than in systemic flares (data not shown). Nevertheless, in light of the large variations in CD177 expression in controls and throughout the patient cohorts, these data have to be interpreted with caution.

This is a small study with a limited number of patients examined longitudinally, even if the results are confirmed in a second cohort. As RNA was extracted from whole blood rather than sorted cells due to the constraints of a biobank, and given that a number of genes discussed, especially CD177, are expressed by neutrophils, changes in neutrophil numbers could have significantly impacted the results. However, not all genes described here are predominantly expressed in neutrophils and CD177 was also consistently upregulated in patients with polyarticular flares and low neutrophil numbers. As variable cell numbers are a valid point of criticism of preceding studies as well, confirmation using sorted cells is a logical next step in researching gene expression patterns in sJIA [4–6]. The strength of this study is the longitudinal examination of patients who have undergone a consistent institutional treatment protocol with IL-1 agonists in different stages of their disease.

## Conclusions

By using this longitudinal analysis, our study identifies novel pathways (STAT4 and BCL6) that might be of relevance in sJIA and indicates strong upregulation of HLA-DRB1 in cooperation with CD74 in patients with sJIA in inactive disease upon treatment with IL-1 antagonists. This provides the first functional confirmation of a previous study, which identified HLA-DRB1 as a risk factor in sJIA. Additionally CD177 was confirmed as a new marker in sJIA. Studies with larger patient cohorts using flow cytometry for protein expression are necessary to confirm these results.

## Additional files

Additional file 1: **Table S1**. List of regulated genes with fold change (FC) > 2 in patients with active disease (ad) versus inactive disease (id) showing signal intensity in both disease states, gene symbol and description. (XLSX 60 kb)

Additional file 2: **Figure S1.** Expression of ANXA3 using RT-PCR: delta Ct values (relative expression values related to RPL) in controls, active systemic or polyarticular disease and inactive disease (***p < 0.001, ****p < 0.0001). **Figure S2.** Expression of IRAK3 using RT-PCR: delta Ct values (relative expression values related to RPL) in controls, active systemic or polyarticular disease and inactive disease (**p < 0.01). (DOCX 619 kb)

## Abbreviations

bp: Base pairs
cDNA: Complementary DNA
Ct: Cycle threshold
HNA-2: Human neutrophil antigen 2
GO: Gene Ontology
IRAK: IL-1 receptor associated kinase
IL: Interleukin
ILAR: International League of Associations for Rheumatology
MHC: Major histocompatibility complex
MIF: Macrophage migration inhibitory factor
PBMC: Peripheral blood mononuclear cells
RA: Rheumatoid arthritis
RPL: Ribosomal protein L
RT-PCR: Reverse transcription PCR
sJIA: Systemic juvenile idiopathic arthritis
TF: Transcription factor
Tfh: Follicular T helper (cells)
