# Supplementary material for: Transcription factor motif enrichment in whole transcriptome analysis identifies STAT4 and BCL6 as the most prominent binding motif in systemic juvenile idiopathic arthritis
Source: Arthritis Res Ther. 2018 May 30;20:98. doi: 10.1186/s13075-018-1603-2 (PMC5977738; doi:10.1186/s13075-018-1603-2)

Additional file 2

Transcription Factor Motif Enrichment in Whole Transcriptome Analysis identifies STAT4 and BCL6 as the Most Prominent Binding Motif in Systemic Juvenile Idiopathic Arthritis

Hügle et al.

**Figure S1:** Expression of ANXA3 using RT-PCR: Delta Ct values (relative expression values related to RPL) in controls, active systemic or polyarticular disease and inactive disease (***p<0.001, ****p<0.0001).

**
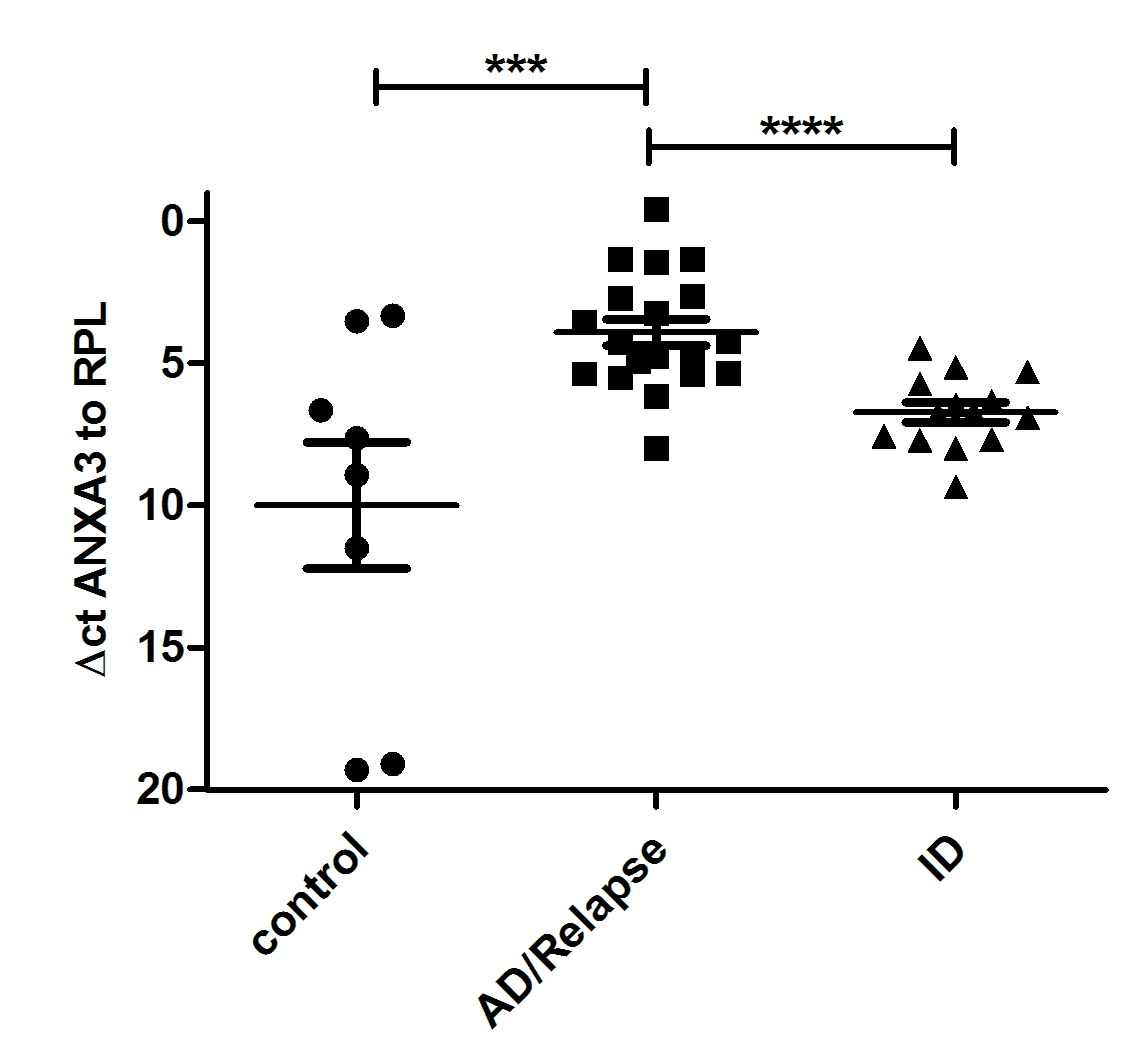
**

**Figure S2:** Expression of IRAK3 using RT-PCR: Delta Ct values (relative expression values related to RPL) in controls, active systemic or polyarticular disease and inactive disease (**p<0.01).


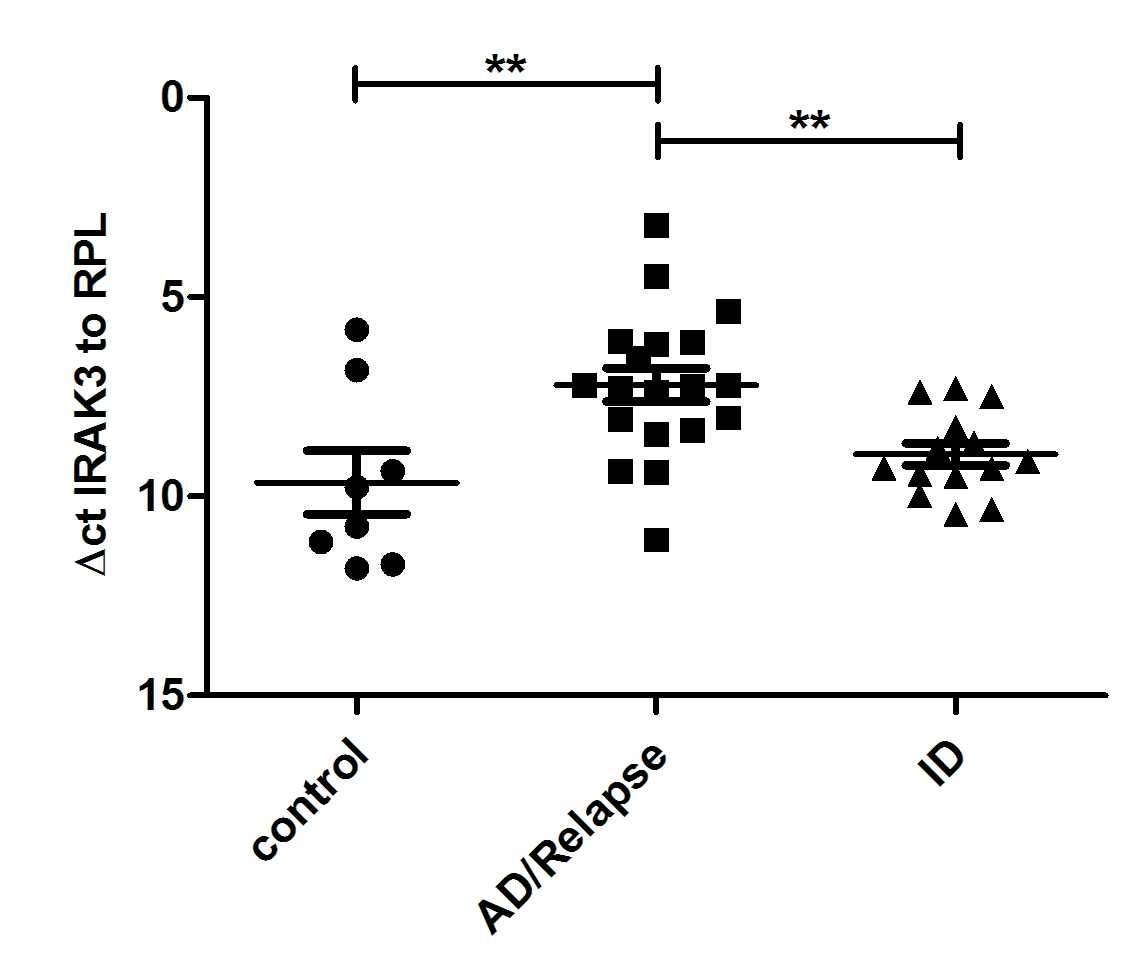

Supplement: Supplementary file 2 — Figure S1. Expression of ANXA3 using RT-PCR: delta Ct values (relative expression values related to RPL) in controls, active systemic or polyarticular disease and inactive disease (***p < 0.001, ****p < 0.0001). Figure S2. Expression of IRAK3 using RT-PCR: delta Ct values (relative expression values related to RPL) in controls, active systemic or polyarticular disease and inactive disease (**p < 0.01). (DOCX 619 kb) [file 13075_2018_1603_MOESM2_ESM.docx]
